# Optimizing Antifungal Use Through Interdisciplinary Intervention in the Hematology Unit

**DOI:** 10.3390/jof12020127

**Published:** 2026-02-11

**Authors:** Maria Alejandra Caro Flautero, Maria Camila Rubio, Edgar Fabián Manrique-Hernández, Jeimy Lorena León, Olga Daniela Vega Jiménez, Paola Cristina Álvarez Mantilla, Pilar Rivas-Pinedo, Alejandra Mendoza-Monsalve, Maricel Licht-Ardila, Alexandra Hurtado-Ortiz, Carlos Augusto Solórzano

**Affiliations:** 1 Department of Immunology, Microbiology and Parasitology, Faculty of Medicine and Nursing, University of the Basque Country (UPV/EHU), Bizkaia Campus, 48940 Leioa, Spain; mcarof@unal.edu.co; 2 Hospital Internacional de Colombia HIC-Fundación Cardiovascular de Colombia FCV, Km 7 Autopista Bucaramanga—Piedecuesta Valle de Mensulí, Piedecuesta 680001, Colombia; marcamilarubio240@gmail.com (M.C.R.); fabianmh1993@gmail.com (E.F.M.-H.); odanielavega@gmail.com (O.D.V.J.); paolaalvarez0330@gmail.com (P.C.Á.M.); mmendoza806@unab.edu.co (A.M.-M.); mlichtar@gmail.com (M.L.-A.); csolorzanor@unal.edu.co (C.A.S.); 3 Medical and Diagnostic Mycology Group, Department of Microbiology, Faculty of Medicine, Universidad Nacional de Colombia, Bogotá 111321, Colombia; jprivasp@unal.edu.co; 4 School of Medicine, Faculty of Medicine, Universidad El Bosque, Bogotá 110121, Colombia; jelolego@gmail.com; 5 Postgraduate Department in Infectious Disease, Universidad de Santander, Bucaramanga 680003, Colombia

**Keywords:** antifungal agents, drug utilization review, antimicrobial stewardship, communicable diseases, hematology

## Abstract

Invasive fungal infections are frequent complications in patients with hematologic malignancies due to immunosuppression and intensive treatments. In Colombia, limited diagnostic availability, heterogeneous prescribing practices, and emerging antifungal resistance highlight the need for optimized use. We evaluated an interdisciplinary antifungal stewardship intervention in the hematology unit of a tertiary-care hospital. A quasi-experimental before-and-after study included 353 hospitalized patients receiving systemic antifungals between 1 January 2023 and 31 December 2024 (1154 prescriptions). Following the intervention, antifungal prescribing shifted toward increased prophylaxis and reduced therapeutic use, with substantial reduction in prophylactic Amphotericin B dosing, stable treatment dosing, and selective changes in agent choice, including decreased voriconazole and discontinuation of some broad-spectrum drugs. Microbiological sampling decreased, reflecting a more targeted diagnostic approach rather than improved documentation. Antifungal consumption patterns showed redistribution among agents rather than uniform reduction. Prophylaxis-related costs increased, while treatment-related costs decreased without statistical significance. ICU admissions and in-hospital mortality remained unchanged. These results demonstrate that structured antifungal stewardship programs are feasible and safe in hematology units in middle-income settings, supporting more rational antifungal use without compromising patient outcomes.

## 1. Introduction

Invasive fungal infections (IFIs) represent a major complication in patients with haematologic malignancies, largely due to disease-related immunosuppression and the intensive treatments required, including chemotherapy and haematopoietic stem cell transplantation [1]. Globally, the incidence of IFIs in patients with acute myeloid leukemia (AML) and myelodysplastic syndromes (MDS) has been reported to reach up to 26.1%, with invasive aspergillosis as the most frequent entity, followed by candidemia and fusariosis [2]. Despite advances in antifungal therapy, the widespread use of empirical treatment, initiation of antifungals without microbiological confirmation, and prolonged therapy in the absence of clear indications have contributed to inappropriate antifungal use, with relevant clinical, microbiological, and economic consequences [3].

In Latin America, and particularly in Colombia, these challenges are compounded by limitations in diagnostic availability, heterogeneous prescribing practices, and restricted access to structured antifungal stewardship resources. Antifungal stewardship programs are relatively underdeveloped in the region, and literature on optimized antifungal use is limited, reflecting barriers in diagnostics and program implementation in resource-constrained settings [4]. Regional evidence also indicates that inappropriate antifungal prescriptions may be highly prevalent, with some studies reporting that up to 72% of antifungal formulations are inappropriate, increasing the risk of adverse events and interactions [5]. In Colombia, surveys of clinical practice highlight weaknesses in antifungal prescribing knowledge and practice among physicians, further underscoring the need for strengthened education and stewardship strategies [6].

Within this regional framework, data from Colombia illustrate the clinical consequences of these challenges. Studies conducted in Bogotá have reported a 30-day mortality of approximately 50% among patients with haematologic malignancies who develop candidemia, alongside a predominance of non-*albicans Candida* species, which are more frequently associated with complex antifungal susceptibility profiles [7]. In parallel, the emergence and sustained transmission of *Candida auris* since its first detection in the country in 2016 has become a major public health concern. National surveillance reports have documented high levels of resistance to fluconazole and Amphotericin B among *C. auris* isolates, underscoring the importance of rational antifungal use in conjunction with strengthened microbiological surveillance and diagnostic stewardship [8]. This evolving resistance landscape further complicates empirical antifungal therapy and highlights the clinical consequences of indiscriminate antifungal exposure.

Importantly, local epidemiology may differ from national surveillance trends. In our institution, the fungal epidemiological profile is driven primarily by *Candida tropicalis* and *Candida albicans*. Among these isolates, fluconazole resistance rates of 12.7% for *C. tropicalis* and 5.4% for *C. albicans* have been observed in recent years. While these findings provide relevant insight into local susceptibility patterns, our institutional microbiological surveillance remains ongoing, and continued data collection will be essential to improve the precision and representativeness of these estimates (Supplementary Table S1). Within hematology units, several specific gaps in antifungal use have been consistently described in the literature [9,10]. These include prolonged empirical antifungal therapy in the absence of microbiological confirmation, delayed or low-yield diagnostic sampling, and the use of broad-spectrum or high-toxicity antifungal agents without standardised dosing strategies or clearly defined indications [5]. Such practices increase the risk of adverse events and hinder the development of reliable institutional epidemiological data to guide clinical decision-making [11].

Antifungal stewardship programmes (AFSPs) have emerged as an effective strategy to optimise antifungal prescribing, dosing, and treatment duration, particularly when supported by coordinated collaboration between haematologists, infectious diseases specialists, microbiologists, and clinical pharmacists. Through structured clinical evaluation, appropriate interpretation of diagnostic tests, and evidence-based therapeutic recommendations, AFSPs aim to promote the rational use of antifungal agents, with infectious diseases specialists often playing a central coordinating role [12,13].

Although AFSPs have demonstrated benefits in high-income settings, such as reduced antifungal toxicity, improved adherence to clinical guidelines, and optimisation of antifungal exposure, evidence from middle-income countries, and from hematology units in Latin America in particular, remains limited. This gap highlights the need for locally generated data assessing the feasibility and impact of AFSP implementation on prescribing practices, patient safety, and resource utilisation in these settings [14]. By optimising antifungal use, stewardship interventions may contribute not only to improved clinical outcomes but also to healthcare system sustainability through reductions in adverse events, length of hospital stay, and associated costs [8,15].

In this context, the present study aimed to evaluate the impact of an interdisciplinary antifungal stewardship intervention implemented in the hematology unit of a tertiary-care hospital in Colombia. The primary objectives were to assess changes in antifungal prescribing patterns, dosing strategies, diagnostic practices, antifungal consumption, and associated costs following the intervention.

## 2. Materials and Methods

A quasi-experimental before-and-after study was conducted to evaluate the impact of an interdisciplinary antifungal stewardship program led by the Infectious Diseases (ID) service on antifungal prescribing practices in the hematology unit of a tertiary care hospital. This institution serves as a referral center for the management of hematological diseases and hematopoietic progenitor cell transplantation in Santander, Colombia. The study was carried out between 1 January 2023, and 31 December 2024, and comprised two phases: a pre-intervention phase (1 January 2023–31 March 2024) and an intervention phase (1 April–31 December 2024).

The study population included patients hospitalized in the hematology unit who had a documented prescription and administration of at least one systemic antifungal agent in their electronic medical records during their hospital stay. Exclusion criteria were hospital stays of less than 48 h, antifungal prescriptions intended exclusively for outpatient care, and incomplete medical records. Antifungal exposure was evaluated at the prescription level, whereas patient-related variables, including sex, age, relapse status, survival at discharge, type of neoplastic disease, receipt of chemotherapy, and length of hospital stay (days), were assessed at the individual patient level. The primary outcome was the change in antifungal prescribing patterns. Secondary outcomes included changes in antifungal dosing strategies, diagnostic practices related to fungal infections, antifungal consumption, and associated costs.

The intervention consisted of an interdisciplinary antifungal stewardship strategy led by the ID service and involved two ID specialists: one responsible for daily consultation activities and on-demand evaluations, and another overseeing a structured weekly antifungal stewardship round (PROA). All hospitalized hematology patients receiving systemic antifungals were screened daily through electronic medical record review and bedside assessment. In addition, a dedicated weekly round evaluated all ongoing antifungal prescriptions using a literature review of indication, diagnostic workup, drug selection, dosing, drug–drug interactions, toxicity, and duration of therapy. Appropriateness of antifungal use was defined according to institutional clinical protocols derived from ECIL, IDSA, and ESCMID guidelines and required adherence to recommended diagnostic evaluation, agent selection based on risk stratification and clinical syndrome, optimized dosing, and timely de-escalation or discontinuation when predefined criteria were met [16,17].

All ID recommendations were mandatory for implementation. In cases of disagreement between the hematology and ID services, a formal interdisciplinary case discussion was convened to reach consensus and document the final therapeutic plan. The intervention was further supported by a pharmacy-managed antifungal restriction and alert system, whereby all prescriptions of broad-spectrum antifungal agents, including liposomal Amphotericin B, Amphotericin B deoxycholate, echinocandins, and mould-active azoles, required verbal or written pre-authorization by an ID specialist before dispensing. The pharmacy system generated real-time alerts for non-authorized prescriptions, prompting immediate ID evaluation; this system was not in place during the pre-intervention phase. IFIs were classified according to the 2020 European Organization for Research and Treatment of Cancer/Mycoses Study Group Education and Research Consortium (EORTC/MSGERC) definitions of proven, probable, and possible IFI. Breakthrough IFI was defined as any proven or probable IFI occurring during ongoing systemic antifungal therapy [18].

Clinical and microbiological data were collected from electronic medical records and the pharmacy service database, and recorded in a structured database designed for the study. Variables included: age, sex, type of hematologic neoplasm, presence of relapse, total length of stay, survival status at discharge, and whether chemotherapy was administered. Regarding antifungal prophylaxis, the following data were documented: type of use, antifungal agent, dose (mg/day), and occurrence of neutropenia at the initiation of prophylaxis. For antifungal treatment, data included use, antifungal agent, dose, and treatment dates. Additionally, it was recorded whether there was clinical suspicion of an invasive fungal infection, the suspected site, whether microbiological samples were taken, and the type of sample. It was also noted whether the patient required ICU admission due to IFI and whether there was an evaluation by infectious diseases or AFSP during hospitalization. Discharge condition was also recorded. The costs of antifungal therapy for both prophylaxis and treatment were also included in the analysis, pre-intervention, and during the intervention. All variables were measured using appropriate qualitative and quantitative scales based on their type.

### Statistical Analysis

A descriptive analysis was performed to summarize the demographic, clinical, and laboratory characteristics of the study population. A mixed unit-of-analysis approach was applied: antifungal exposure and utilization variables were analyzed at the prescription level, recognizing that individual patients could receive more than one antifungal agent during hospitalization, whereas demographic and clinical variables were analyzed at the patient level.

Normality of continuous variables was assessed using the Shapiro–Wilk test. Variables with a normal distribution were expressed as means with standard deviations, while non-normally distributed variables were reported as medians with interquartile ranges. Categorical variables were summarized as absolute frequencies and percentages. Comparisons between the pre-intervention and intervention phases were performed using the Mann–Whitney U test for continuous variables and the Chi-square or Fisher’s exact test for categorical variables, as appropriate. All statistical tests were two-sided, and a *p* value < 0.05 was considered statistically significant. A cost analysis was conducted to assess the economic impact and efficiency of the intervention. All statistical analyses were performed using Stata version 16.

## 3. Results

The study included 353 patients, of whom 224 were in the pre-intervention phase and 129 in the intervention phase. The majority of patients were male in both periods (54.46% pre-intervention vs. 59.69% intervention), and the median age was similar between groups (57 vs. 55 years). Regarding underlying hematologic malignancies, non-Hodgkin lymphoma was the most frequent diagnosis in both cohorts, followed by acute myeloid leukemia and acute lymphoblastic leukemia. The proportion of patients receiving chemotherapy associated with a high risk of bone marrow aplasia was comparable between periods, as was hospital length of stay. No statistically significant differences were observed across baseline demographic or clinical characteristics, indicating well-balanced pre-intervention and intervention groups (Table 1).

Overall, the intervention period was characterized by a shift toward a more prophylaxis-oriented antifungal strategy, with a marked reduction in therapeutic use (from 13.80% to 6.17%) and optimization of dosing practices. Despite a significantly higher proportion of patients presenting with neutropenia during the intervention (30.89% vs. 14.83%), the need for antifungal treatment decreased, and both suspected infectious foci and microbiological sampling became less frequent. Prophylactic Amphotericin B dosing was substantially reduced (median 350 mg to 50 mg), whereas treatment dosing patterns remained largely unchanged (Table 2).

Regarding microbiological diagnostics, samples were obtained from 234 patients during the study period. These included 195 cultures and additional non-culture-based diagnostic tests, comprising molecular assays, antigen detection, and histopathological studies. Only 11 cultures yielded positive results, corresponding to 7 patients. Three isolates were recovered from non-sterile site secretions; the remaining isolates were obtained from blood cultures. Identified species included *Candida albicans* (*n* = 1), *Candida glabrata* (*n* = 1), *Candida krusei* (*n* = 2), *Candida parapsilosis* (*n* = 1), and *Candida tropicalis* (*n* = 6), reflecting a predominance of non-*albicans Candida* species among culture-positive cases. With respect to additional indirect, non-culture-based diagnostic tests, 38 samples yielded positive results for *Aspergillus* spp. through polymerase chain reaction (PCR) and galactomannan antigen detection, highlighting the relevance of mould diagnostics within the institutional epidemiological profile, particularly in the haematology setting (Table 2).

Additionally, ICU admission occurred in 6.81% of patients during the pre-intervention phase and in 5.82% during the intervention period (*p* = 0.488). In the cost analysis, expenditures on antifungal prophylaxis increased from USD 82,177 in the pre-intervention period to USD 115,109 during the intervention (*p* < 0.001), reflecting a deliberate shift toward broader prophylactic coverage and optimization of preventive use. In contrast, treatment-related costs decreased from USD 198,593 in the pre-intervention period to USD 138,048 during the intervention (*p* = 0.498), suggesting a reduced need for therapeutic antifungal use. Taken together, these findings indicate that the intervention was associated with a reallocation of resources from treatment to prevention, without relevant changes in ICU admission rates (Table 2).

**Figure 1:**
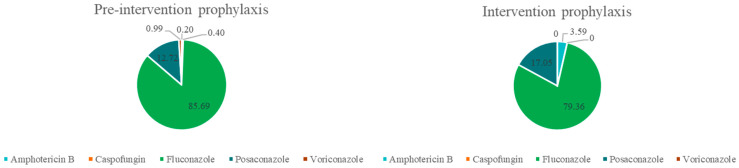
Percentage distribution of antifungal agents prescribed for prophylaxis in hospitalized hematology patients before and during the antifungal stewardship intervention. Hospitalized adult patients with hematologic diseases admitted to a tertiary-care hospital in Colombia during the pre-intervention (1 January 2023–31 March 2024; *n* = 224) and intervention (1 April–31 December 2024; *n* = 129) periods; pie charts represent the percentage (%) distribution of antifungal agents used for prophylaxis.

During the pre-intervention period, antifungal prophylaxis was largely dominated by fluconazole, with posaconazole used in a smaller proportion of cases. Following the intervention, fluconazole remained the cornerstone of prophylactic therapy, although its relative use decreased modestly, reflecting a more selective prescribing approach. Notably, the use of broader-spectrum agents such as caspofungin and voriconazole was completely discontinued during the intervention phase (*p* < 0.001). Overall, these changes indicate a shift toward a more focused and standardized prophylactic strategy, prioritizing fluconazole and minimizing unnecessary exposure to broader-spectrum antifungal agents (Figure 1).

**Figure 2:**
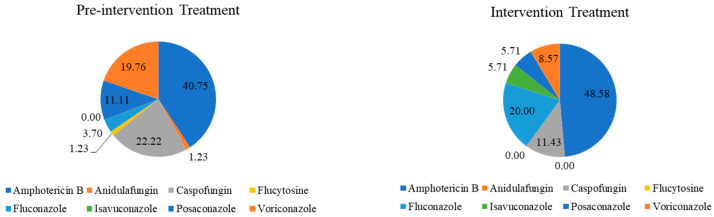
Percentage distribution of antifungal agents used for treatment in hospitalized adult patients with hematologic diseases before and during the intervention period. Hospitalized adult patients with hematologic diseases admitted to a tertiary-care hospital in Colombia during the pre-intervention (1 January 2023–31 March 2024) and intervention (1 April–31 December 2024) periods; pie charts represent the percentage (%) distribution of antifungal agents used for treatment.

During the pre-intervention period, antifungal treatment relied on a broad range of agents, with Amphotericin B as the predominant option, followed by echinocandins and voriconazole. After implementation of the stewardship intervention, treatment practices became more streamlined, with a greater concentration on Amphotericin B and fluconazole and the incorporation of isavuconazole in selected cases. In contrast, the use of echinocandins, voriconazole, and posaconazole declined markedly, and some agents were no longer prescribed. Overall, these changes reflect a consolidation of antifungal treatment strategies during the intervention period, favoring a narrower and more standardized therapeutic approach (Figure 2).

In the comparison of antifungal prophylaxis days between the pre-intervention and intervention periods, no statistically significant difference was observed (*p* = 0.737). The median days of prophylaxis for Amphotericin B decreased from 17 (IQR 17–17) days in the pre-intervention phase to 9 (IQR 5–14) days during the intervention. Caspofungin maintained a constant median of 2 (IQR 2–2) days in both periods. Fluconazole showed similar medians, 9 (IQR 5–21) days before and 9.5 (IQR 5–20) days in the intervention. Posaconazole presented a median of 15 (IQR 6–31) days pre-intervention and 12 (IQR 6–28) days during the intervention. Voriconazole use was stable at 8 (IQR 7–13) days in both phases.

**Figure 3:**
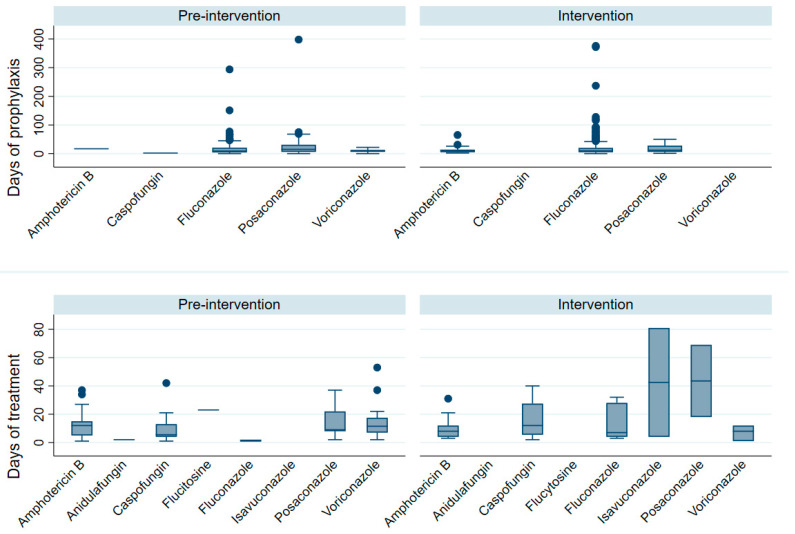
Duration of Antifungal Prophylaxis and Treatment Before and During the Intervention. Hospitalized adult patients with hematologic diseases admitted to a tertiary-care hospital in Colombia during the pre-intervention (1 January 2023–31 March 2024) and intervention (1 April–31 December 2024) periods; boxplots display the duration (days) of antifungal prophylaxis and treatment by agent, showing medians and interquartile ranges.

For antifungal treatment duration, the analysis also revealed no significant differences between periods (*p* = 0.988). Amphotericin B decreased slightly from 11.5 (IQR 4.5–15) to 8 (IQR 4–12) days. Anidulafungin remained unchanged at 2 (IQR 2–2) days. Caspofungin increased from 5.5 (IQR 4–13) to 12 (IQR 5.5–27.5) days. Flucytosine remained constant at 23 (IQR 23–23) days. Fluconazole increased from 1 (IQR 1–2) to 7 (IQR 4–28) days. Posaconazole rose markedly in the intervention period to 43.5 (IQR 18–69) days from 9 (IQR 8–22) days pre-intervention. Voriconazole showed a median decrease from 11.5 (IQR 7–17.5) to 8 (IQR 1–12) days. Isavuconazole was only recorded in the intervention period, with a median of 42.5 (IQR 4–81) days (Figure 3). Antifungal durations were generally comparable between periods, with observed differences confined to specific agents rather than a uniform increase in treatment duration.

**Figure 4:**
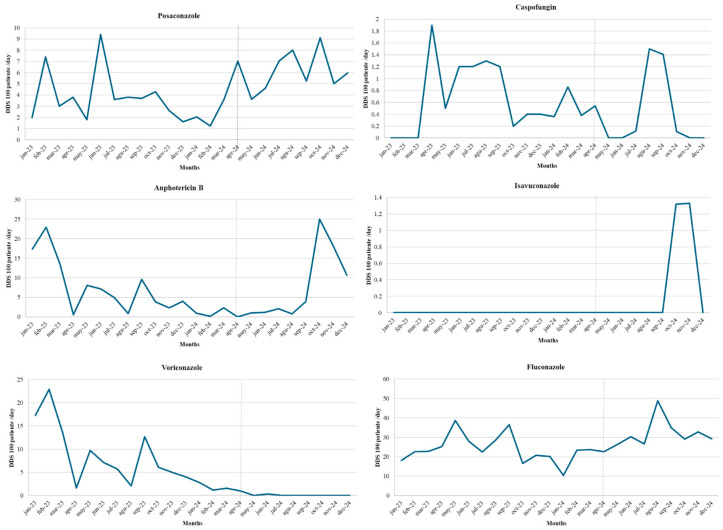
Antifungal Consumption During Pre-Intervention and Intervention Periods. Hospitalized adult patients with hematologic diseases admitted to a tertiary-care hospital in Colombia during the pre-intervention (1 January 2023–31 March 2024) and intervention (1 April–31 December 2024) periods; line graphs show monthly antifungal consumption expressed as defined daily doses per 100 bed-days (DDD/100 bed-days) by agent.

During the pre-intervention period, the monthly consumption of antifungal agents, expressed in DDD/100 bed-days, showed considerable variability across active ingredients, with values ranging from 0 to 38.6. In this period, fluconazole, voriconazole, and Amphotericin exhibited the highest consumption, with marked fluctuations and peaks in different months, showing pronounced increases at the beginning of the observation period and a decreasing trend for Amphotericin and voriconazole. In contrast, posaconazole, caspofungin, and isavuconazole displayed low or intermittent values, with notable isolated increases such as that of posaconazole in June 2023 (9.4) and isavuconazole in June 2023 (3.1). During the intervention period, a heterogeneous pattern was observed; for most antifungal agents, consumption levels were lower compared to the pre-intervention period, except for Amphotericin and fluconazole, which showed peaks of 24.98 and 48.83 DDD/100 bed-days, respectively, between August and October 2024. Voriconazole and isavuconazole, in turn, maintained values close to zero. In the analysis of differences between groups before and during the intervention, statistically significant differences were observed for posaconazole (*p* = 0.005), fluconazole (*p* = 0.015), and voriconazole (*p* < 0.001). In contrast, no significant differences were found for caspofungin (*p* = 0.263) or Amphotericin B (*p* = 0.742). Taken together, these findings indicate that the intervention was associated with a more selective and restrained antifungal consumption profile, with reductions concentrated in azoles other than fluconazole (Figure 4).

## 4. Discussion

The implementation of an interdisciplinary antifungal stewardship program in the hematology unit was associated with statistically significant changes in antifungal use patterns and diagnostic practices, demonstrating its effectiveness in optimizing the use of these agents. In the pre and during intervention comparative analysis, an increase in the proportion of prophylactic prescriptions was observed, along with a reduction in therapeutic indications. The observed changes in antifungal prescribing reflect not only improved adherence to institutional protocols and international guideline recommendations but also a broader cultural shift toward evidence-based antifungal use, facilitated by consistent stewardship input and close coordination with hematology teams [19]. The structured involvement of infectious diseases (ID) specialists appears to have strengthened clinicians’ confidence in guideline-concordant prescribing, supporting the discontinuation of antifungal agents with limited evidence for routine use. This process represents a recognized quality indicator of effective stewardship programs, in which sustained behavioral change relies not only on written protocols but also on trust in specialist expertise and real-time clinical support [10]. Similar findings have been reported in other settings, where successful antifungal stewardship interventions were associated with active specialist involvement, high prescribing volume, and access to timely diagnostics, ultimately contributing to the standardization of practices and long-term changes in clinical behavior [10,19].

These principles are reinforced by the 2024 ESCMID European guidelines and the ECIL initiative, both of which underscore that stewardship effectiveness relies on close collaboration among clinicians, infectious disease specialists, and microbiologists [20]. In Latin America, such programs remain scarce; however, a study conducted in Brazil demonstrated a reduction in inappropriate antifungal prescriptions from 80.2% to 64.6% following an educational intervention [21]. The present study provides evidence from Colombia, where no prior formal publications on structured antifungal stewardship interventions in hematology were available.

One of the most notable stewardship-related effects observed during the intervention period was the substantial reduction in the median prophylactic dose of Amphotericin B. This change is unlikely to be explained solely by differences in baseline patient characteristics and more plausibly reflects stewardship-driven optimization of dosing practices. The structured involvement of infectious disease specialists facilitated adherence to evidence-based dosing recommendations and discouraged the use of unnecessarily high prophylactic doses. Given the well-established dose-dependent nephrotoxicity and electrolyte disturbances associated with Amphotericin B, dose optimization represents a clinically meaningful stewardship outcome. Importantly, this reduction occurred without a concomitant increase in mortality or ICU admission rates, suggesting that lower prophylactic dosing was achieved without compromising patient safety. Nevertheless, due to the observational nature of this study, causality cannot be inferred [22]. In parallel, the use of agents without current justification as first-line prophylactic options (such as caspofungin and voriconazole) was eliminated.

A redistribution of prophylactic agents was also observed: although fluconazole remained predominant, its relative use decreased, while posaconazole increased. These changes are consistent with the literature, which positions posaconazole as superior to fluconazole in patients with acute leukemia and allogeneic transplantation, given its association with reduced incidence of IFI and related mortality. The continued use of fluconazole at a relevant proportion may be explained by local epidemiology, characterized by a low incidence of aspergillosis and a high prevalence of *Candida* spp., including *C. auris*, for which fluconazole efficacy may vary depending on susceptibility patterns [23]. This consideration is particularly relevant in the Colombian context. While in Europe and North America the replacement of fluconazole with posaconazole is nearly universal among high-risk patients, in regions with a lower prevalence of aspergillosis, the risk–benefit balance may differ. Consequently, the coexistence of these agents as valid prophylactic options reflects an approach tailored to local epidemiological realities, representing an additional methodological strength of the program [23,24].

With regard to diagnostic practices, a reduction in microbiological sampling was observed during the intervention period, together with a decrease in cases classified as having presumed infectious foci. Although this finding may initially appear counterintuitive in the context of antifungal stewardship, it should not be interpreted as improved diagnostic adherence or accuracy, as diagnostic yield, timing, and appropriateness were not directly assessed. Rather, these observations likely reflect a more targeted, clinically driven diagnostic approach, supported by stewardship input and the application of standardized diagnostic criteria, which may have facilitated the reclassification of situations previously considered presumptive [14]. The involvement of ID specialists may have improved clinical discrimination, reducing the overinterpretation of nonspecific findings and focusing microbiological investigations on scenarios more likely to influence management decisions. This interpretation remains hypothetical and should be approached with caution. While international guidelines increasingly emphasize targeted diagnostic strategies supported by biomarkers such as galactomannan, PCR, and beta-D-glucan [22,25], not all of these tools are widely available in Colombian hospitals. Nevertheless, the present study suggests that meaningful improvements in diagnostic rationalization can still be achieved through structured clinical evaluation and selective use of available microbiological studies. Future studies incorporating diagnostic performance indicators and microbiological yield are needed to better characterize the impact of antifungal stewardship interventions on diagnostic practices [13].

Interpretation of the study findings must also account for the higher proportion of neutropenic patients during the intervention period. Neutropenia is a key clinical confounder that may independently influence antifungal prescribing patterns, including the decision to initiate prophylaxis, agent selection, and treatment duration. Despite this higher-risk clinical profile, no deterioration in key clinical outcomes was observed, supporting the safety of stewardship-driven antifungal optimization even in a more vulnerable population [24].

Regarding overall antifungal consumption, expressed in DDD/100 bed-days, statistically significant differences were observed for posaconazole, fluconazole, and voriconazole, demonstrating a tangible impact of the program on utilization pressure. The absence of significant changes in Amphotericin B and caspofungin consumption likely reflects variability in specific clinical needs and the limited sample size of these subgroups [5,12]. Analysis of antifungal consumption revealed a redistribution of antifungal exposure rather than a uniform reduction. Notably, the decreased use of voriconazole is clinically relevant, as this agent is frequently employed empirically in high-risk febrile patients and is associated with a well-recognized risk of hepatotoxicity and clinically significant drug–drug interactions. Limiting unnecessary exposure to voriconazole may therefore contribute to improved patient safety while also reducing the use of a comparatively high-cost antifungal agent [26].

From an economic perspective, antifungal prophylaxis costs increased significantly during the intervention period, reflecting the intentional expansion and optimization of prophylactic strategies, including greater use of mould-active agents in selected high-risk patients. In contrast, treatment-related antifungal costs showed a numerical decrease, although this reduction did not reach statistical significance. Collectively, these findings suggest a redistribution of resource utilization from treatment toward prevention rather than a net reduction in overall antifungal expenditure [15]. Although a formal cost-effectiveness analysis was beyond the scope of this study, the observed shift toward preventive antifungal use, together with reduced voriconazole exposure and lower prophylactic doses of Amphotericin B, may serve as indirect indicators of improved efficiency and more rational allocation of antifungal resources [27].

From a public health perspective, such optimization is relevant not only for institutional sustainability but also for reducing unnecessary antifungal exposure at the population level, potentially mitigating selective pressure and the emergence of antifungal resistance. In resource-limited settings, antifungal stewardship interventions may therefore support more equitable allocation of high-cost therapies, preservation of antifungal effectiveness, and improved preparedness for emerging resistant pathogens. This economic and public health dimension is particularly relevant in middle-income healthcare systems, where stewardship programs must balance clinical benefit with sustainable resource utilization [28,29,30].

Regarding clinical outcomes, no significant differences were observed in in-hospital mortality or ICU admission rates between the pre-intervention and intervention periods. Although these findings do not demonstrate a survival benefit, they provide reassurance that stewardship-driven changes in antifungal selection, dosing, and utilization did not compromise patient safety. The absence of a measurable reduction in overall mortality also highlights the inherent limitations of single-center studies with limited sample sizes and the use of all-cause mortality as an outcome. As reported in previous antifungal stewardship studies, benefits are more consistently observed in prescribing quality, antifungal exposure, and resource utilization, whereas an impact on mortality generally requires larger, multicenter cohorts or infection-attributable outcomes [31]. These considerations underscore the need for collaborative multicenter initiatives in Colombia and Latin America to more accurately evaluate the clinical impact of antifungal stewardship interventions.

In summary, this study provides real-world evidence that a structured antifungal stewardship program implemented in a hematology unit can effectively modify daily clinical practice by optimizing antifungal prophylaxis, improving dosing strategies, and promoting more rational use of diagnostic resources without compromising patient safety. The observed changes in prescribing patterns and antifungal utilization occurred without significant differences in mortality or other critical outcomes, supporting the feasibility and effectiveness of the intervention in a high-risk hematology population.

Some limitations should be acknowledged, including the single-center, quasi-experimental before–after design, which limits generalizability and does not fully exclude residual confounding. Additionally, infection-attributable outcomes, diagnostic yield, and formal cost-effectiveness analyses were not assessed. Nevertheless, these limitations do not detract from the relevance of the findings, as this study represents one of the first reports of antifungal stewardship implementation in hematology in Colombia and contributes contemporary data from a middle-income setting with a substantial burden of IFIs. Future research should prioritize multicenter studies to enhance statistical power and generalizability, incorporate diagnostic performance metrics and microbiological resistance surveillance, particularly regarding emerging pathogens such as *Candida auris*, and include formal economic evaluations. Finally, as no multivariable adjustments or interrupted time-series analyses were performed, residual temporal confounding cannot be completely excluded; therefore, some of the observed changes may be influenced by underlying trends not directly attributable to the intervention [32].

## 5. Conclusions

The implementation of an interdisciplinary antifungal stewardship program in a hematology unit was associated with meaningful improvements in prescribing quality, including a shift toward prophylaxis-oriented strategies, optimization of prophylactic Amphotericin B dosing, and discontinuation of antifungal agents no longer supported for routine use. These changes were achieved without adverse effects on key clinical outcomes, supporting the safety of stewardship-driven optimization in a high-risk population.

Overall, the intervention promoted a more selective and clinically driven use of diagnostics and a redistribution of antifungal exposure from treatment to prevention, without increasing total antifungal consumption. These findings demonstrate that antifungal stewardship programs are feasible and effective in hematology units in middle-income settings and represent a pragmatic strategy to optimize antifungal use while preserving patient safety.

## Figures and Tables

**Table 1:** Demographic and Clinical Characteristics of Patients Before and During the Intervention Period.

Variable	Category	Pre-Intervention (*n* = 224) *n* (%)	Intervention (*n* = 129) *n* (%)	Total	*p*-Value
Sex	Female	102 (45.54)	52 (40.31)	154 (43.63)	0.340
	Male	122 (54.46)	77 (59.69)	199 (56.37)	
Age *		57 (26–67)	55 (40–66)	56 (41–67)	0.590
Relapse	Yes	50 (22.32)	28 (21.71)	78 (22.10)	0.893
	No	174 (77.68)	101 (78.29)	275 (77.90)	
Survival status at discharge	Alive	194 (86.61)	115 (89.15)	309 (87.54)	0.487
	Deceased	30 (13.39)	14 (10.85)	44 (12.46)	
Neoplastic disease	Hemolytic anemia	0 (0.00)	2 (0.89)	2 (0.57)	0.482
	Aplasia Medular	5 (3.91)	6 (2.68)	11 (3.13)	
	Plasma cell leukemia	0 (0.00)	2 (0.89)	2 (0.57)	
	Acute lymphoblastic leukemia	19 (14.84)	35 (15.63)	54 (15.34)	
	Chronic lymphocytic leukemia	0 (0.00)	4 (1.79)	4 (1.14)	
	Acute myeloid leukemia	20 (15.63)	38 (16.96)	58 (16.48)	
	Chronic myeloid leukemia	4 (3.13)	6 (2.68)	10 (2.84)	
	Promyelocytic leukemia	2 (1.56)	6 (2.68)	8 (2.27)	
	Hodgkin’s lymphoma	9 (7.03)	17 (7.59)	26 (7.39)	
	Non-Hodgkin’s lymphoma	56 (43.75)	84 (37.50)	140 (39.77)	
	Mycosis fungoides	0 (0.00)	1 (0.45)	1 (0.28)	
	Multiple myeloma	4 (3.13)	16 (7.14)	20 (5.68)	
	High-risk MDS	4 (3.13)	1 (0.45)	5 (1.42)	
	Intermediate-risk MDS	5 (3.91)	5 (2.23)	10 (2.84)	
	Granulocytic sarcoma	0 (0.00)	1 (0.45)	1 (0.28)	
Chemotherapy	High risk of bone marrow aplasia	117 (52.00)	61 (50)	178 (51.3)	0.737
	No high risk of bone marrow aplasia	108 (48.00)	61 (50)	169 (48.7)	
Hospital length of stay (days)		27.5 (15–58)	30 (17–51)	29 (16–53)	0.989

Hospitalized adult patients with hematologic diseases admitted to the hematology unit of a tertiary-care hospital in Colombia during the pre-intervention (1 January 2023–31 March 2024) and intervention (1 April–31 December 2024) periods. Columns present absolute numbers and percentages [*n* (%)] or * median and interquartile range (IQR), as appropriate.

**Table 2:** Comparison of Antifungal Use Patterns, Dosages, and Clinical Characteristics Before and During Intervention.

Variable	Category	Pre-Intervention (*n* = 587) *n* (%)	Intervention (*n* = 567) *n* (%)	Total (*n* = 1154) *n* (%)	*p*-Value
Type of use	Treatment	81 (13.80)	35 (6.17)	116 (10.05)	<0.001
	Prophylaxis	506 (86.20)	532 (93.83)	1038 (89.95)	
Daily dose (mg/day) in prophylaxis *	Amphotericin B	350 (350–350)	50 (44–63)	50 (44.5–131.5)	<0.001
	Caspofungin	50 (50–50)	–	50 (50–50)	
	Fluconazole	200 (200–200)	200 (200–400)	200 (200–200)	
	Posaconazole	300 (300–300)	300 (300–300)	300 (300–300)	
	Voriconazole	200 (200–400)	–	200 (200–400)	
Daily dose (mg/day) in treatment *	Amphotericin B	83 (50–200)	190 (64–380)	156 (55–290)	0.142
	Anidulafungin	100 (100–100)	–	100 (100–100)	
	Caspofungin	50 (50–50)	50 (50–50)	50 (50–50)	
	Flucytosine	7000 (7000–7000)	–	7000 (7000–7000)	
	Fluconazole	800 (800–800)	400 (200–400)	400 (400–800)	
	Posaconazole	300 (300–300)	300 (300–300)	300 (300–300)	
	Voriconazole	400 (200–400)	200 (200–400)	400 (200–400)	
	Isavuconazole	–	200 (200–200)	200 (200–200)	
Neutropenia	Yes	86 (14.83)	173 (30.89)	259 (22.72)	<0.001
Suspected focus	Yes	102 (17.41)	58 (10.23)	160 (13.88)	<0.001
Microbiological sampling performed	Yes	157 (26.79)	77 (13.60)	234 (20.31)	<0.001
ICU admission	Yes	40 (6.81)	33 (5.82)	73 (6.33)	0.488
Cost in prophylaxis	USD	$82,177	$115,109	$197,285	<0.001
Cost of treatment	USD	$198,593	$138,048	$336,641	0.498

Hospitalized adult patients with hematologic diseases admitted to the hematology unit of a tertiary-care hospital in Colombia during the pre-intervention (1 January 2023–31 March 2024) and intervention (1 April–31 December 2024) periods. Data are presented as *n* (%) or * median (interquartile range). ICU: Intensive Care Unit; USD: United States dollars.

## Data Availability

Data are available from the corresponding author upon reasonable request due to privacy and ethical restrictions.

## Supplementary Materials

The following supporting information can be downloaded at: https://www.mdpi.com/article/10.3390/jof12020127/s1, Table: S1. Antifungal resistance profile of Candida species.
