# Supplementary material for: Optimizing Antifungal Use Through Interdisciplinary Intervention in the Hematology Unit
Source: J Fungi (Basel). 2026 Feb 11;12(2):127. doi: 10.3390/jof12020127 (PMC12942500; doi:10.3390/jof12020127)
Supplement: Supplementary file 1 [file jof-12-00127-s001.zip › jof-4017267-supplementary.pdf]

**Supplementary Table S1.** Antifungal resistance profile of *Candida* species.

| ***Candida* species (n)** | **Fluconazole (%R)** | **Voriconazole (%R)** | **Caspofungin (%R)** | **Micafungin (%R)** |
|---|---|---|---|---|
| ***C. albicans* (56)** | 5.36 | 0.00 | 3.57 | 1.79 |
| ***C. glabrata* (18)** | NA | NA | NA | 0.00 |
| ***C. parapsilosis* (17)** | 5.88 | 0.00 | 0.00 | 0.00 |
| ***C. tropicalis* (55)** | 12.70 | 7.27 | 0.00 | 0.00 |
| ***C. auris* (9)** | NA | NA | NA | NA |
| ***C. guilliermondii* (1)** | NA | NA | NA | 0.00 |
| ***C. haemulonii* (1)** | NA | NA | NA | NA |
| ***C. krusei* (3)** | NA | 0.00 | 0.00 | 0.00 |

**Note:** *Candida* species were isolated from hospitalized patients in a tertiary-care hospital in Colombia between January 2023 and December 2024. %R, percentage of resistant isolates; NA, not available (susceptibility testing not performed or insufficient isolates).
